# Fluoroquinolone Amorphous Polymeric Salts and Dispersions for Veterinary Uses

**DOI:** 10.3390/pharmaceutics11060268

**Published:** 2019-06-09

**Authors:** Hanah Mesallati, Anita Umerska, Lidia Tajber

**Affiliations:** 1 School of Pharmacy and Pharmaceutical Sciences, Trinity College Dublin, College Green, 2 Dublin, Ireland; mesallah@tcd.ie (H.M.); umerskam@tcd.ie (A.U.); 2 SSPC, Synthesis and Solid State Pharmaceutical Centre, Limerick, Ireland; 3 MINT, UNIV Angers, INSERM 1066, CNRS 6021, Universite Bretagne Loire, 4 rue Larrey, CEDEX, 49933 Angers, France

**Keywords:** enrofloxacin, ciprofloxacin, amorphous solid dispersion, amorphous polymeric salt, polymer, ball milling, solubility, dissolution

## Abstract

Enrofloxacin (ENRO) is a poorly soluble drug used in veterinary medicine. It differs from the more widely used fluoroquinolone ciprofloxacin (CIP) by the presence of an ethyl substituent on its piperazine amino group. While a number of recent studies have examined amorphous composite formulations of CIP, little research has been conducted with ENRO in this area. Therefore, the main purpose of this work was to produce amorphous solid dispersions (ASDs) of ENRO. The solid-state properties of these samples were investigated and compared to those of the equivalent CIP ASDs, and their water uptake behavior, solubility, dissolution, and antibacterial activity were assessed. Like CIP, X-ray amorphous solid dispersions were obtained when ENRO was ball milled with acidic polymers, whereas the use of neutral polymers resulted in semi-crystalline products. Proton transfer from the carboxylic acids of the polymers to the tertiary amine of ENRO’s piperazine group appears to occur in the ASDs, resulting in an ionic bond between the two components. Therefore, these ASDs can be referred to as amorphous polymeric salts (APSs). The glass transition temperatures of the APSs were significantly higher than that of ENRO, and they were also resistant to crystallization when exposed to high humidity levels. Greater concentrations were achieved with the APSs than the pure drug during solubility and dissolution studies, and this enhancement was sustained for the duration of the experiments. In addition, the antimicrobial activity of ENRO was not affected by APS formation, while the minimum inhibitory concentrations and minimum bactericidal concentrations obtained with the APS containing hydroxypropyl methylcellulose acetate succinate grade MG (HPMCAS-MG) were significantly lower than those of the pure drug. Therefore, APS formation is one method of improving the pharmaceutical properties of this drug.

## 1. Introduction

**Figure 1:**
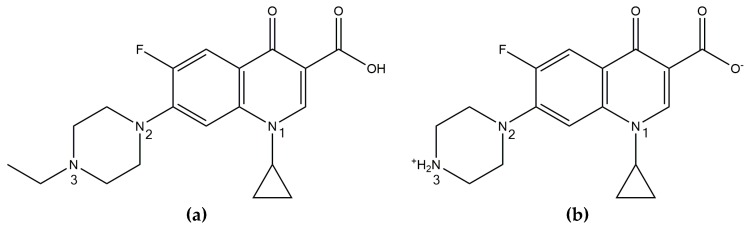
Chemical structure of (**a**) enrofloxacin and (**b**) ciprofloxacin.

Enrofloxacin (ENRO), or 1-cyclopropyl-7-(4-ethylpiperazin-1-yl)-6-fluoro-4-oxo-1,4-dihydroquinoline-3-carboxylic acid, is a fluoroquinolone antibiotic that is licensed for veterinary use. ENRO differs from the more widely known fluoroquinolone ciprofloxacin (CIP) by the presence of an ethyl substituent in the N3 position (Figure 1). Anhydrous CIP generally exists in the zwitterionic state, with a protonated amino group and negatively charged carboxylate group. These oppositely charged groups form head-to-tail ionic bonds with neighboring and adjacent molecules, resulting in a tetramer-like structure [1]. ENRO, on the other hand, is unionized in the solid state and can therefore only form a number of weak C–H•••O and C–H•••N hydrogen bonds [2].

CIP and ENRO are both poorly water-soluble drugs, with an intrinsic solubility of approximately 0.1 mg/mL and 0.4 mg/mL, respectively [3]. Both drugs are least soluble at pH 7.4 and exist predominantly in the zwitterionic form in neutral solutions [4]. Despite the theoretically higher hydrophilicity of CIP due to the absence of an aliphatic group in the N3 position, the strong crystal lattice of this drug reduces its aqueous solubility below that of ENRO. In addition, the extra ethyl group of ENRO increases its lipophilicity and permeability, resulting in greater absorption in rat in situ permeability studies than CIP [3]. However, the permeability of ENRO still falls within the limits of poorly permeable [5].

One of the most commonly used techniques to improve the solubility of ionizable drugs is salt formation. A number of crystalline ENRO salts have been produced by Karanam et al. and all were found to be significantly more water-soluble than the pure drug [2]. The piperazine N3 nitrogen of ENRO is positively charged in the salts containing acidic counterions, such as succinic acid, fumaric acid, and maleic acid, and forms an ionic bond with the carboxylate groups of the acids. The carboxylic acid of the drug, on the other hand, remains unionized. By contrast, ENRO exists in the anionic state in the ammonium salt, with a negatively charged carboxylate group and neutral piperazine group [2]. The solubility of ENRO was also increased significantly via formulation as the saccharinate salt. Like the equivalent CIP salt, an ionic interaction between the negatively charged saccharin molecule and positively charged N3 amino group of ENRO was detected in this compound [6].

The solubility of a drug may also be increased by converting it to the amorphous form. This involves the disruption of the crystal lattice, producing a disordered, high-energy version of the drug [7]. This approach is usually avoided during commercial drug development due to the intrinsic instability of amorphous solids. However, suitable excipients can be used to stabilize the amorphous form and prevent its crystallization. This stabilization is usually brought about via interactions between the components, such as hydrogen or ionic bonds, and/or through steric hindrance, e.g., by polymers with long chains [8]. Recently, the formation of various amorphous solid dispersions (ASDs) and amorphous salts of CIP was investigated by our group. Due to the poor solubility and thermal degradation of the drug, these were mainly prepared by ball milling [1,9]. Promising results were obtained with a number of CIP ASDs containing various acidic polymers. They were found to increase the glass transition temperature (*T*_g_) and solubility of CIP, while the permeability and antibacterial activity of the drug was either unchanged or moderately improved [9]. As an ionic interaction between the drug and polymer was identified in each as these ASDs, they may also be referred to as amorphous polymeric salts (APSs) [9]. The majority of ASDs described in the literature are stabilized by nonionic interactions between the components, such as hydrogen bonds, and do not involve proton transfer between the drug and polymer. The apparent solubility of APSs may be even further improved in comparison to unionized ASDs, due to the amalgamation of both approaches, i.e., drug ionization and amorphization.

Amorphous salts of CIP containing succinic acid or amino acids as counterions have also been prepared. While these formulations were far more soluble than the CIP ASDs, they were less stable when exposed to high humidity and in most cases decreased the permeability of the drug [10,11].

Unlike many other poorly soluble drugs, there is very little in the literature regarding amorphous solid dispersions of ENRO, and no mention of the pure amorphous form of the drug. However, one study by Chun and Choi described the preparation of an enrofloxacin–Carbopol “complex” by mixing a solution of ENRO in 1% acetic acid with that of Carbopol in water, filtering and washing the precipitate, and then drying and milling the resultant powder [12]. The product was found to be X-ray amorphous but lacked a clear *T*_g_. The authors hypothesized that the positively charged tertiary amine of the drug formed an ionic bond with the carboxylate anions of Carbopol. Consequently, when the dissolution rate of the complex was found to be lower than that of the pure drug, this was attributed to the strength of the drug–polymer interactions. 

Due to the absence of research in this area, the main aim of this project was to prepare ASDs of ENRO and to examine their solid-state and pharmaceutical properties. As previously mentioned, the chemical structure of ENRO differs from that of CIP by the presence of an ethyl group on its N3 piperazine amino group. It was of interest to determine whether this has an impact on the interactions that the drug can form with various polymers and whether the biopharmaceutical properties of this veterinary drug can be improved. The solid-state characteristics and water uptake behavior of the successfully prepared dispersions were also examined and compared to those of equivalent CIP ASDs produced in an earlier study [9]. In addition, the solubility, dissolution, and antibacterial activity of the ENRO dispersions were investigated in order to determine the effect of physicochemical transformations on these biopharmaceutical properties of the drug.

## 2. Materials and Methods 

### 2.1. Materials

Enrofloxacin (ENRO) was obtained from Glentham Life Sciences (Wiltshire, UK) and Ciprofloxacin (CIP) was purchased from Carbosynth Limited (Berkshire, UK). Polyvinylpyrrolidone K17 (PVP: Plasdone C-15) was sourced from ISP Technologies (New Jersey, USA), poly(vinyl alcohol) (PVA: 98% hydrolyzed, Mw 13000–23000) was obtained from Sigma-Aldrich (St. Louis, MO, USA), and Carbopol 981 was purchased from BF Goodrich (OH, USA). Methacrylic acid methyl methacrylate copolymer (Eudragit L100) and methacrylic acid ethyl acrylate copolymer (Eudragit L100-55) were kindly donated by Evonik Röhm GmbH (Darmstadt, Germany), while hydroxypropyl methylcellulose acetate succinate grades LG and MG (HPMCAS-LG and HPMCAS-MG) were provided by Shin-Etsu Chemical Co., Ltd. (Tokyo, Japan). 

Fasted state simulated intestinal fluid (FaSSIF) was produced by adding 2.24 g SIF^®^ Powder Original (biorelevant.com, Surrey, UK) to one liter of FaSSIF phosphate buffer, consisting of 19.5 mM NaOH (Riedel-de Haën, Seelze, Germany), 25 mM NaH_2_PO_4_·H_2_O (Merck, Darmstadt, Germany) and 106 mM NaCl (Sigma-Aldrich Ireland Ltd., Arklow, Ireland), adjusted to pH 6.5 with NaOH. Triethylamine was obtained from Sigma-Aldrich Ireland Ltd., (Arklow, Ireland). Brain–heart infusion (BHI) broth was obtained from bioMérieux (Marcy l’Étoile, France), while plates with Columbia agar supplemented with sheep blood were sourced from Oxoïd (Dardilly, France). All other chemicals and solvents were of analytical grade.

### 2.2. Methods

#### 2.2.1. Sample Preparation

Solid dispersions were produced by dry ball milling ENRO and various polymers as described previously [9]. Briefly, the process was carried out at room temperature (22–25 °C) with a Retsch planetary ball mill PM 100 (Haan, Germany). The polymer concentration used was 40–60% (*w*/*w*), and a total of 2 g of powder was loaded to 50 mL stainless steel grinding jars containing three 20 mm stainless steel milling balls. Each mixture was milled for 1–6 h in total, in intervals of 15 min with 10 min breaks in between. Crystalline ENRO was quench cooled by heating the drug to the endset of melting (~235 °C) at 10 °C/min in a differential scanning calorimetry (DSC) machine, and then immediately removing the sample to allow it to cool quickly at room temperature. Physical mixtures (PMs) were prepared by mixing relevant concentrations of ENRO and the polymers in a pestle and mortar for a few minutes.

#### 2.2.2. Powder X-ray Diffraction (PXRD)

PXRD was performed at room temperature using a benchtop Rigaku MiniflexII X-ray diffractometer (Tokyo, Japan) and a Haskris cooler (Illinois, USA) as described previously [1]. 

#### 2.2.3. Solid-State Fourier Transform Infrared Spectroscopy (FTIR)

A Spectrum One FTIR spectrometer (PerkinElmer, Connecticut, USA) was utilized to obtain FTIR data [1,9]. The following parameters of the analysis, accumulating 10 scans in total, were employed: 450–4000 cm^−1^ was spectral range, 4 cm^−1^ was resolution, while the scan speed was 0.2 cm/s. A sample concentration of 1% (*w*/*w*) was obtained, diluting the powdered sample with KBr and making disks suitable for FTIR by applying pressure of approximately 10 bar for 1 min. 

Deconvolution of the FTIR spectra was conducted to facilitate their comparison. OriginPro 7.5 software was used to subtract the baseline and carry out Gaussian peak fitting on the spectra. In each case, seven overlapping peaks were detected in the region under examination, whose combined area and shape were similar to those of the original bands [1].

#### 2.2.4. Differential Scanning Calorimetry (DSC)

DSC analysis on 5–10 mg samples was done using a Mettler Toledo DSC (Schwerzenbach, Switzerland) under nitrogen purge and employing sealed 40 µL aluminum pans with three pin-holes in the lid [9]. To expose the glass transition temperature (*T*_g_) of the samples, the powders were first subjected to a first heating cycle from 25 to 65 °C to remove the residual moisture, then the samples were cooled to 25 °C and re-heated to 250 °C at a rate of 10 °C/min. 

#### 2.2.5. Modulated Temperature Differential Scanning Calorimetry (MTDSC)

The *T*_g_s of the ASDs were detected by MTDSC using a Q200 DSC instrument and TA Instruments DSC Refrigerated Cooling System (TA Instruments, New Castle, Delaware). Samples of 3–4 mg were heated in aluminum pans with sealed aluminum lids. Nitrogen was used as the purge gas at a flow rate of 20 mL/min. Samples were heated from 0 °C to 110–185 °C at 2 °C/min, with an amplitude of ± 0.318 °C and a modulation period of 60 s. Results were analyzed with the Universal Analysis 2000 software (TA Instruments). The midpoint of the transition was taken as the *T*_g_. Sapphire was used to calibrate the heat capacity, while indium was used for the calibration of enthalpy and temperature. All measurements were carried out in triplicate.

#### 2.2.6. Calculation of Theoretical Glass Transition (*T*_g_) Values with Gordon–Taylor Equation

The theoretical *T*_g_s of the ASDs were calculated using the Gordon–Taylor equation [13,14]:

(1) $$T_{g}=\frac{w_{1}T_{g1}+Kw_{2}T_{g2}}{w_{1}+Kw_{2}},$$

where *K* is approximately equal to:

(2) $$K\approx \frac{T_{g1}\rho_{1}}{T_{g2}\rho_{2}}.$$

*w*_1_ and *w*_2_ are the weight fractions of the components, *T*_g1_ and *T*_g2_ are the glass transition temperatures of ENRO and the polymer, and ρ_1_ and ρ_2_ are the true densities of the two constituents. The *T*_g_s of the polymers were sourced from the literature: HPMCAS-LG, 119 °C [15]; HPMCAS-MG, 120 °C [15]; Eudragit L100, 130 °C [16]; Eudragit L100-55, 96 °C [16]; and Carbopol, 105 °C [17]. Further, the average true density data were obtained from the published resources: ENRO, 1.385 g/cm^3^ [18]; HPMCAS-LG and HPMCAS-MG, 1.29 g/cm^3^ [19]^;^ Eudragit L100, 0.84 g/cm^3^ [20]; Eudragit L100-55, 0.83 g/cm^3^ [16]; and Carbopol, 1.4 g/cm^3^ [21].

#### 2.2.7. High-Speed Differential Scanning Calorimetry (HSDSC)

HSDSC was performed on crystalline ENRO, under helium purge, with a PerkinElmer Diamond DSC (Waltham, MA, USA) supported by a ULSP B.V. 130 cooling system (Ede, The Netherlands) as described previously [1]. Around 3–5 mg samples were first encapsulated in aluminum pans (18 µL) and heated from 25 to 300 °C at a rate of 300–500 °C/min. 

#### 2.2.8. Thermogravimetric Analysis (TGA)

TGA was done using a Mettler TG50 measuring module coupled to a Mettler Toledo MT5 balance (Schwerzenbach, Switzerland) [1]. The heating rate employed was 10 °C/min and samples (8–10 mg) were loaded into open aluminum pans. 

#### 2.2.9. Dynamic Vapor Sorption (DVS) and Mathematical Modeling Using Young–Nelson Equations

DVS studies were performed using an Advantage-1 automated gravimetric vapor sorption analyzer (Surface Measurement Systems Ltd., London, UK) at 25.0 ± 0.1 °C, between 0 and 90% RH, in steps of 10% RH, as described previously [9]. The complete sorption and desorption profile is shown as an isotherm. PXRD analysis was performed on all samples following DVS to identify any solid-state transformations.

In order to determine how water uptake occurs in the ASDs, the experimental sorption and desorption data were fitted to equations using the Young–Nelson model, as described previously [22,23]:

M_s_ = A(β + θ) + B(θ)RH, (3)

M_d_ = A(β + θ) + B(θ)RH_max_. (4)

M_s_ and M_d_ are the amount of water sorbed and desorbed, respectively, at each relative humidity value. This is expressed as a fraction of the dry mass of the sample. A and B are constants which can be defined as follows:

(5) $$A=\frac{\rho_{w}Vol_{M}}{W_{m}},$$

(6) $$B=\frac{\rho_{w}Vol_{A}}{W_{m}}.$$

ρ_*w*_ is the density of water, *W_m_* is the weight of the dry sample, and *Vol_M_* and *Vol_A_* are the volume of adsorbed and absorbed water, respectively. In Equations (3) and (4), θ represents the fraction of sample surface that is covered by at least one layer of water molecules, and β is the mass of absorbed water at 100% RH. B(θ)RH is therefore the mass of absorbed water at a particular fraction of monolayer coverage, θ, and RH level. A(β + θ) is equal to the total amount of adsorbed water, while Aθ is the mass of water in an entire adsorbed monolayer, as a fraction of the dry mass of the material. Aβ is the mass of water adsorbed in a multilayer. θ and β may be further defined as follows [23]:

(7) $$\theta =\frac{\mathrm{RH}}{\mathrm{RH}+E\left(1-\mathrm{RH}\right)},$$

(8) $$\beta =-\frac{\mathrm{ERH}}{E-\left(E-1\right)\mathrm{RH}}+\frac{E^{2}}{\left(E-1\right)}\ln a\frac{E-\left(E-1\right)\mathrm{RH}}{E}a-\left(E+1\right)\ln\left(1-\mathrm{RH}\right).$$

E is an equilibrium constant between water in the monolayer and condensed water adsorbed externally to the monolayer:

(9) $$E=e^{-\left[\frac{q_{1}-q_{L}}{k_{B}T}\right]}.$$

q_1_ is the heat of adsorption of water on the solid, q_L_ is the heat of condensation of water, both in Joules/mole, then T is the temperature in Kelvin and k_B_ is Boltzmann’s constant (1.38 × 10^−23^ J/K).

The experimental data obtained from DVS studies of the ENRO ASDs, as well as equivalent CIP ASDs that were previously prepared [9], were fitted to Equations (3) and (4) by iterative multiple linear regression. The sum of the squares of the residuals between the experimental and calculated values was used as fitting criteria. The multiple correlation coefficient (r) was calculated using Microsoft Excel 2007. Using the calculated values of A, B, θ, and β, the profiles of water adsorbed in monolayer (Aθ) and multilayer (Aβ), and of absorbed water (Bθ) were determined [23].

#### 2.2.10. Dynamic Solubility Study

A volume of 5 mL of FaSSIF was added to 40 mL glass vials and placed into jacketed beakers connected to a Lauda M12 waterbath at 37 °C (Lauda-Königshofen, Germany). A quantity of pure drug or ASD, in excess of the expected saturated solubility (25–200 mg, depending on the sample), was added to the vials containing the aliquot of FaSSIF and stirred at 1000 rpm. At different time points, over a 2 h period, samples were taken for the stirred suspensions and filtered with 0.45 µm PTFE membrane filters (VWR, USA). The filtered solutions were then diluted appropriately with a 2.9 g/L solution of phosphoric acid, previously adjusted to pH 2.3 with trimethylamine [2]. The concentration of ENRO in each of the diluted samples was determined by UV spectrophotometry as described below. The solid material left in the vials at the end of the studies was filtered and analyzed by PXRD.

#### 2.2.11. Dissolution Study

Dissolution studies were carried out at 37 °C, using a paddle apparatus (Apparatus II) with a continuous rotation of 100 rpm. A quantity of sample corresponding to approximately 10% of the final drug concentration obtained in the solubility study was added to 300 mL of FaSSIF (ENRO: 25 mg, ENRO/Eudragit L100: 287.5 mg, ENRO/HPMCAS-LG: 967.5 mg and ENRO/HPMCAS-MG: 517.5 mg). One milliliter aliquots was taken at specific time points over the 2 h period of the study and replaced with 1 mL of FaSSIF. Each sample was filtered with a 0.45 µm PTFE membrane filter (VWR, USA) and diluted with a 2.9 g/L solution of phosphoric acid, previously adjusted to pH 2.3 with triethylamine. The concentration of ENRO in each of the diluted samples was then measured by UV spectrophotometry. The cumulative quantity of dissolved drug at each time point was calculated by taking account of the 1 mL aliquots taken for analysis. Each study was carried out in triplicate.

#### 2.2.12. UV Spectrophotometry

UV analysis was performed using a Shimadzu UV-1700 PharmaSpec UV-vis spectrophotometer (Shimadzu Corp., Kyoto, Japan) using quartz cuvettes with a 1 cm optical path length. The reference was a 2.9 g/L solution of phosphoric acid, previously adjusted to pH 2.3 with triethylamine. This buffer was also used to produce a range of concentrations of pure ENRO, in order to construct a calibration curve. The λ_max_ of these solutions was found to be 277 nm; therefore, UV absorbance was measured at this wavelength.

#### 2.2.13. Bacterial Studies

For these studies, *Staphylococcus aureus* ATCC 25923, *Escherichia coli* ATCC 25922, *Pseudomonas aeruginosa* ATCC 27853, and *Klebsiella pneumoniae* DSM 16609 were cultured on Columbia agar supplemented with sheep blood. The inoculum was prepared as previously described [9,24]. The density of the *S. aureus* suspension was adjusted so that it equaled that of the 1.1 McFarland standard, and then further diluted 100-fold with BHI medium. The *P. aeruginosa*, *E. coli*, and *K. pneumoniae* suspensions, on the other hand, were adjusted to equal that of the 0.5 McFarland standard, and then diluted 10-fold.

The minimum inhibitory concentrations (MICs) and minimum bactericidal concentrations (MBCs) of ENRO and the ASDs were determined using a broth microdilution method, as previously described [9,24]. 

## 3. Results and Discussion

### 3.1. Production of Amorphous Solid Dispersions/Amorphous Polymeric Salts

**Figure 2:**
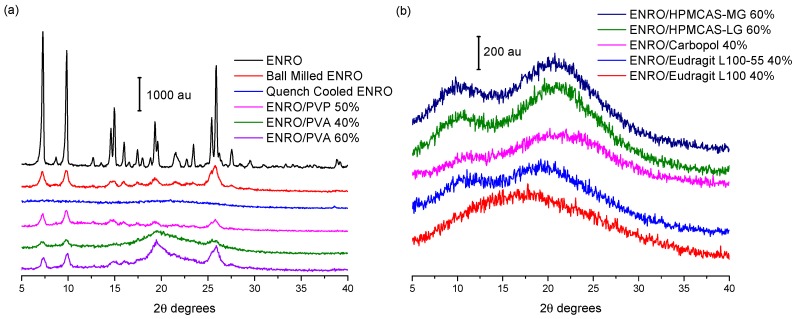
Powder X-ray diffraction (PXRD) diffractograms of (**a**) enrofloxacin (ENRO) and semi-crystalline solid dispersions, and (**b**) ENRO amorphous solid dispersions (ASDs).

Ball milling was first carried out on crystalline “as received” ENRO to determine whether it is possible to amorphize the drug in this manner. However, following four hours of milling at room temperature, a disordered, semi-crystalline solid was obtained (Figure 2a). This was also the case with CIP [1]. The most intense peaks in the X-ray diffractogram of the unprocessed ENRO powder are visible at 7.4, 9.8, 14.9, and 25.8 2θ degrees. These peaks are also present in the diffractogram of ball milled ENRO; however, their intensity is reduced. Quench cooling ENRO, on the other hand, resulted in an X-ray amorphous material (Figure 2a).

In previous studies with CIP, X-ray amorphous solid dispersions were obtained when the drug was ball milled with Eudragit L100, Eudragit L100-55, Carbopol, HPMCAS-LG, and HPMCAS-MG. All of these polymers are acidic, and FTIR analysis confirmed the presence of an ionic bond between the positively charged piperazine amino group of CIP and the carboxylate groups of the polymers in the ASDs [9]. These acidic polymers also proved to be suitable co-formers for ENRO, with each resulting in an X-ray amorphous formulation (Figure 2b). As was the case with CIP, a polymer concentration of 60% (*w*/*w*) was required to fully amorphize mixtures of CIP and HPMCAS, whereas 40% (*w*/*w*) was adequate for Eudragit L100, Eudragit L100-55, and Carbopol. Although the product obtained with 40% (*w*/*w*) HPMCAS-LG was almost X-ray amorphous following 4 h of milling, very small peaks could still be detected by PXRD at 9.8 and 25.8 2θ degrees, corresponding to the most prominent peaks of anhydrous ENRO (Figure S1). A slightly more crystalline product was obtained with HPMCAS-MG under the same conditions, which decreased in intensity following a further 2 h of milling but did not disappear entirely. In contrast to CIP, which required a total of 6 h of milling and a reduced temperature of 2–5 °C to form ASDs with 60% (*w*/*w*) HPMCAS [9], 4 h of milling at room temperature was adequate for the corresponding ENRO ASDs (Figure S1). This indicates that the polymers may interact more readily with ENRO than CIP possibly due to the weaker crystal lattice of ENRO, which would facilitate its amorphization. These results show that the presence of an extra ethyl group in the structure of ENRO does not appear to negatively affect its ability to interact with these acidic polymers. To enable closer comparison with the equivalent CIP ASDs, the ENRO/HPMCAS ASDs containing 60% (*w*/*w*) polymer that were milled for 6 h were used for further studies.

In contrast to the acidic excipients, when CIP was milled with neutral polymers such as PVP and PVA at a concentration of 40–60% (*w*/*w*), a semi-crystalline product was obtained [9]. This was also the case with ENRO (Figure 2a). The fact that X-ray amorphous solid dispersions were only formed when ENRO was milled with acidic polymers containing carboxylic acid groups suggests that the drug is interacting with these substances via ionic bonds, as was the case with CIP. Likewise, in all of the ENRO salts produced by Karanam et al. containing an acidic counterion, proton transfer from the acid to the piperazine tertiary amine (N3) of the drug occurred, resulting in an ionic interaction between the two moieties [2]. A similar reaction may take place between the N3 of ENRO and the polymers in these ASDs.

### 3.2. Solid-State Fourier Transform Infrared Spectroscopy

**Figure 3:**
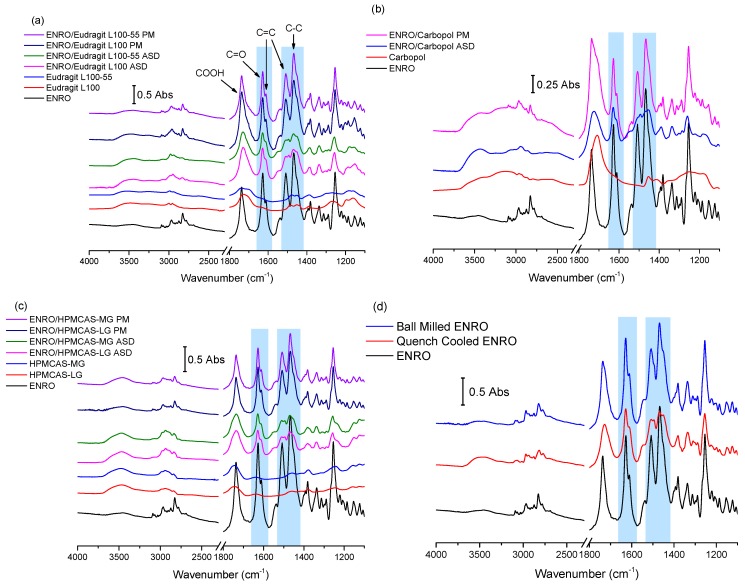
FTIR spectra of ASDs and physical mixtures (PM) containing (**a**) Eudragit L100 and Eudragit L100-55 40% (*w*/*w*) (**b**) Carbopol 40% (*w*/*w*), (**c**) hydroxypropyl methylcellulose acetate succinate grades LG and MG (HPMCAS-LG and HPMCAS-MG) 60% (*w*/*w*), and (**d**) ball milled and quench cooled ENRO. The areas of the spectra that undergo significant changes upon amorphization are highlighted in violet.

The results of FTIR analysis of the ASDs, PMs, and starting materials are shown in Figure 3a–d. A sharp peak is located at 1737 cm^−1^ in the spectrum of crystalline ENRO due to the carbonyl stretch of its unionized carboxylic acid group. While the process of ball milling introduced some disorder to the crystal lattice of ENRO, the FTIR spectrum of the ball milled drug was almost identical to the crystalline ENRO starting material. The greater molecular disorder of quench cooled ENRO, on the other hand, is evident in the broader and less intense peaks of its spectrum (Figure 3d). Slight peak shifts were also seen with this sample, in particular, the carboxylic acid C=O stretch, which shifted to 1728 cm^−1^. This can be attributed to changes in the drug’s intermolecular interactions upon amorphization, such as hydrogen bonding [25]. Interestingly, the COOH carbonyl stretch of the drug also shifted to lower wavenumbers in the spectrum of the crystalline ENRO saccharinate salt, in which the piperazine N3 amino group of the drug is positively charged [6]. This carbonyl peak underwent a similar shift with all of the ASDs. Therefore, while the carboxylic acid of ENRO remains unionized in the ASDs, changes in the hydrogen bonding of this group clearly occur upon amorphization. This shift may also be related to changes in the ionization state of the drug.

The main differences between the spectra of ENRO and the ASDs may be seen in the 1650–1450 cm^−1^ region. In the case of crystalline ENRO, the carbonyl stretch of its ketone group appears as a sharp, strongly absorbing peak at 1628 cm^−1^. The medium intensity shoulder at 1611 cm^−1^ may be assigned to C=C stretching vibrations of the drug’s aromatic ring. While these peaks are not significantly shifted in the spectra of the ASDs, differences in their relative absorbance were observed. In crystalline ENRO, the absorbance of the ketone peak is approximately 1.8 times greater than that of the aromatic peak. This ratio decreases to 1.5–1.7 for each of the ASDs. However, a similar decrease in the relative absorbance of these peaks was also seen with quench cooled ENRO and is therefore likely due to changes in the interactions of these groups upon amorphization.

The peaks at 1508 and 1469 cm^−1^ in the spectrum of ENRO may be attributed to C=C stretching of the aromatic ring, and C–C stretching of the drug’s piperazine group, respectively [26]. The shape of these peaks was altered notably in the ASDs, and the presence of multiple overlapping peaks became evident. In order to separate the individual peaks in this region and to quantify their relative absorbance, deconvolution of the spectra, with Gaussian peak fitting, was carried out. The resulting spectra are shown in Figure S2. Deconvolution allowed the detection of a further peak at approximately 1453 cm^−1^ in the spectrum of ENRO, which may be tentatively assigned to the C–H bending vibrations of the ethyl group. This peak is also present in the spectra of ball milled and quench cooled ENRO, and all of the ASDs, along with an additional peak at approximately 1494 cm^−1^. Although a slight broadening is visible at this wavenumber in the spectrum of crystalline ENRO, it is not as distinct as with the other samples. Clear differences in the relative absorbance of these peaks may also be seen between the pure drug and ASDs. For instance, in crystalline ENRO, the area of the peak at 1508 cm^−1^ is approximately two times smaller than the combined area of the peaks at 1469–1453 cm^−1^. While a similar ratio was obtained with the equivalent peaks in ball milled ENRO, with the quench cooled form of the drug, it decreased to 1.9. With the ASDs, on the other hand, this ratio decreased further to 1.3–1.55. Similarly, in the spectra of crystalline and ball milled ENRO, the absorbance of the peak at 1469 cm^−1^ is clearly greater than that at 1453 cm^−1^. By contrast, in each of the ASDs, as well as quench cooled ENRO, the maximum absorbance of these peaks did not differ greatly. Similar changes in this region were seen in the spectra of the partially crystalline ENRO/PVA solid dispersion, whereas the less disordered ENRO/PVP more closely resembled the crystalline ENRO starting material (Figure S3).

As previously mentioned, the terminal tertiary amine of ENRO (N3) may be protonated in these ASDs, forming ionic bonds with the carboxylate groups of the polymers. If this is the case, the main differences in the FTIR spectra of the ASDs compared to the starting materials or PMs can be attributed to the change in ionization state of the drug, and the presence of an additional ^+^N–H bond. Unfortunately, the ^+^N–H stretch is difficult to assign with certainty, as it will produce a weak band in the 3000–2600 cm^−1^ region that possibly overlaps with others, such as that of the C–H stretch of the neighbouring aliphatic group [26]. Similarly, the ^+^N–H bend of a tertiary amine salt generally appears as a very weak band in the 1610–1500 cm^−1^ region [27] and therefore is likely to be obscured by more intense peaks in the spectra of the ASDs. However, as described above, a number of differences in the 1450–1550 cm^−1^ region of the spectra of ENRO and the ASDs were observed. Therefore, it is possible that the presence of the peak corresponding to the ^+^N–H bend contributed to the variations in this area of the spectra. In addition, as the peaks in this region correspond to groups surrounding the terminal amino group of ENRO, it is likely that they would be altered upon the protonation of N3.

The hypothesis that ENRO is protonated in these ASDs is supported by the FTIR analysis of ENRO salts conducted by Karanam et al. [2]. In the spectra of each of the salts containing an acidic counterion, a decrease in the absorbance of the peak around 1469 cm^−1^ relative to that at 1508 cm^−1^ can be seen, in common with the ENRO ASDs. Single crystal X-ray diffraction confirmed that the N3 of the drug was protonated in these salts and formed an ionic bond with the carboxylate groups of the acids. Therefore, it is likely that ENRO is in the same cationic state in these ASDs and interacts with the acidic groups of the polymers to form amorphous polymeric salts (APSs). The fact that the spectrum of quench cooled ENRO is similar to that of the ASDs may be due to the partial conversion of the drug to the zwitterion. 

### 3.3. Thermal Analysis

**Figure 4:**
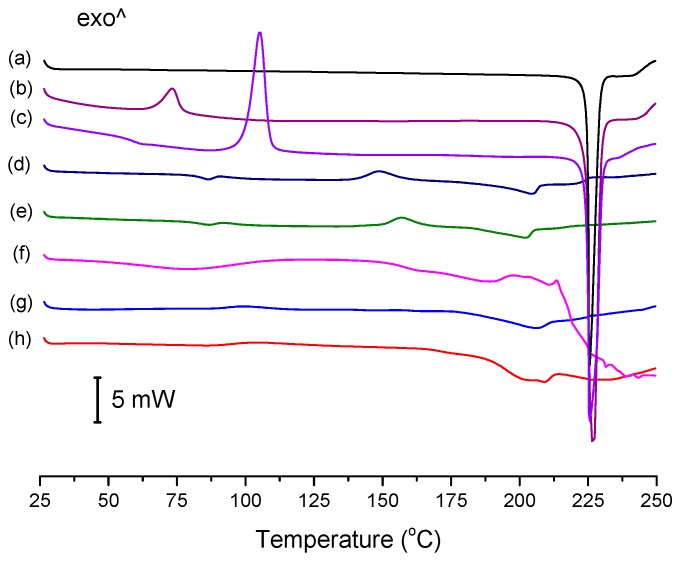
Differential scanning calorimetry (DSC) thermograms of (a) crystalline ENRO, (b) ball milled ENRO, (c) quench cooled ENRO, (d) ENRO/HPMCAS-MG, (e) ENRO/HPMCAS-LG, (f) ENRO/Carbopol, (g) ENRO/Eudragit L100-55, and (h) ENRO/Eudragit L100. The thermograms of the ASDs are those obtained from the second heating cycle, following initial heating to 65 °C to allow for residual water removal.

The conventional DSC thermograms of ENRO and the ASDs are shown in Figure 4. The melting point onset of crystalline ENRO, as well as the ball milled and quench cooled drug, was approximately 225 °C. Its lower melting point in comparison to CIP (approximately 272 °C) [1] can be explained by the less extensive intermolecular bonds in ENRO. In contrast to the pure drug, the thermograms of the ASDs were missing a clear melting point. Similarly, the ASDs did not show distinct crystallization exotherms during DSC analysis, although the small, broad peaks visible at approximately 157 °C and 148 °C in the thermograms of ENRO/HPMCAS-LG and ENRO/HPMCAS-MG, respectively, may be due to some crystallization. The indistinct nature of the thermograms can be attributed to the amorphous nature of these formulations and their stability upon heating [28]. By contrast, ball milled and quench cooled ENRO had clear crystallization peaks at approximately 73 °C and 106 °C, respectively, confirming their lower resistance to crystallization. The particularly low crystallization temperature of ball milled ENRO is to be expected, as the residual crystallinity present in this sample would enable crystal growth to occur more quickly upon heating. 

The *T*_g_ of quench cooled ENRO was detected at 58.9 °C, which is significantly lower than that of CIP (86.7 °C). Again, this may be attributed to the weaker intermolecular interactions present in ENRO. As a distinct *T*_g_ could not be found for all of the ENRO ASDs using conventional DSC; they were therefore analyzed by MTDSC. The resultant *T*_g_s are listed in Table 1. In each case, a single *T*_g_ was detected. This suggests that the drug is miscible with each of these polymers, and that they form a single homogeneous phase [29]. Due to its low amorphous content and high crystallization rate, no *T*_g_ could be determined for ball milled ENRO with either DSC technique. 

The Gordon–Taylor (G–T) equation was used to calculate the expected *T*_g_s of the ASDs, given their weight percentage of drug and polymer. From Table 1, it can be seen that the experimental *T*_g_s of the ASDs containing Carbopol, Eudragit L100, and Eudragit L100-55 were substantially higher than the theoretically derived values. Such large positive deviations from the predicted *T*_g_s suggest that strong interactions exist between the components and are particularly indicative of polymeric salt formation [30]. By contrast, the experimental and G–T *T*_g_s of the HPMCAS-containing ASDs differed by only a few degrees. This suggests that these polymers are fully miscible with ENRO but do not form strong interactions with the drug or that the heteromolecular drug–polymer interactions may be of a similar strength to the homomolecular interactions present in the individual raw materials [31]. 

Similar results have been obtained with the equivalent CIP ASDs, whereby the experimental *T*_g_s of those containing Eudragit L100 and L100-55 deviated from the predicted values by a much greater degree than those containing either grade of HPMCAS. The apparently weaker interactions present in the latter ASDs were attributed to the lower proportion of carboxylic acid groups present in HPMCAS compared to the other polymers [9]. This would explain why a polymer concentration of 60% (*w*/*w*) was required to produce X-ray amorphous solid dispersions with these polymers, whereas a concentration of 40% (*w*/*w*) was sufficient with the others.

As previously mentioned, CIP exists as a zwitterion in the solid state, with a positively charged piperazine amino group and negatively charged carboxylate group. However, it has been shown to convert to the unionized form upon melting, due to intramolecular proton transfer. This was visualized as a small endothermic peak in the DSC thermogram of the drug, just prior to the melting endotherm. However, this low energy event was only visible when CIP was heated at 500 °C/min [1]. HSDSC analysis was therefore carried out on crystalline ENRO in order to determine if it also undergoes proton transfer at high temperatures, in this case from the unionized form to the zwitterion. However, even when heated at the maximum heating rate of 500 °C/min, the drug did not show any evidence of solid-state transformation (Figure S4). After heating ENRO to the endset of melting and allowing it to cool slowly, PXRD and FTIR analysis confirmed that the drug remained in the unionized anhydrous state; thus, the ethyl moiety attached to N3 prevented the proton transfer. 

**Figure 5:**
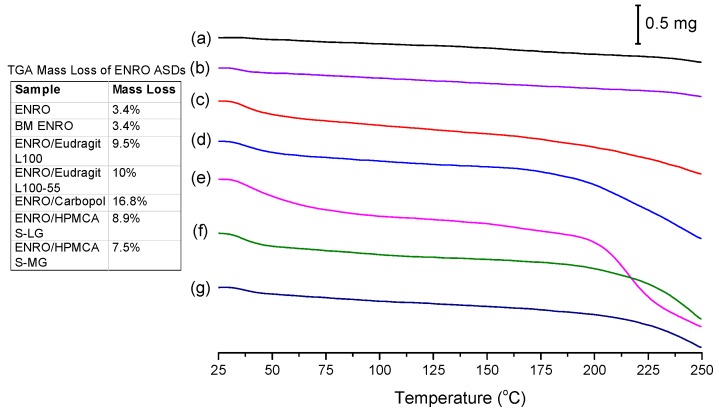
TGA analysis of (a) crystalline ENRO, (b) ball milled (BM) ENRO, (c) ENRO/Eudragit L100, (d) ENRO/Eudragit L100-55, (e) ENRO/Carbopol, (f) ENRO/HPMCAS-LG, and (g) ENRO/HPMCAS-MG.

While crystalline ENRO is pale yellow, quench cooled ENRO is a more vibrant golden color, and when heated to 250 °C, the drug becomes dark orange/rusty. CIP also turns from off-white to a yellow color prior to melting; however, when heated past its melting point, it becomes brown due to substantial degradation. From the TGA curves obtained with ENRO and the ASDs (Figure 5), crystalline and ball milled ENRO do not appear to undergo substantial thermal degradation, decreasing in mass by only 3.4% over the course of the TGA analysis. CIP, on the other hand, is much more prone to thermal degradation, with a mass loss of 12.8% and 17.3% being obtained with the crystalline and ball milled forms of the drug, respectively [1]. An initial mass loss was observed below 70 °C with all of the amorphous formulations due to water evaporation. This is to be expected with ASDs, as the hygroscopic nature of amorphous drugs and polymers results in the absorption of atmospheric water vapor. The amorphous samples also degraded to a greater degree than the pure drug, in particular, the Carbopol ASD. Amorphous solids are typically more reactive than their crystalline counterparts, as their higher molecular mobility can enable such degradation reactions to occur [32]. Alternatively, this mass loss may simply be due to degradation of the polymers.

### 3.4. Water Sorption Studies

**Figure 6:**
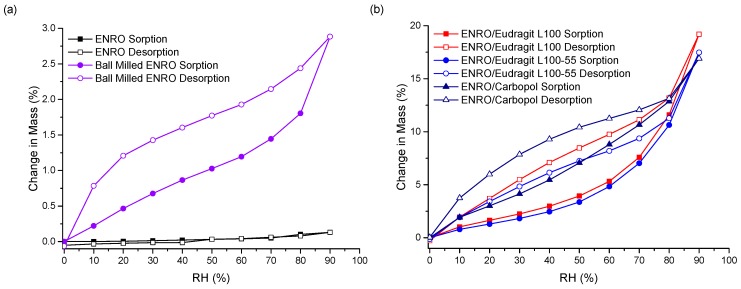

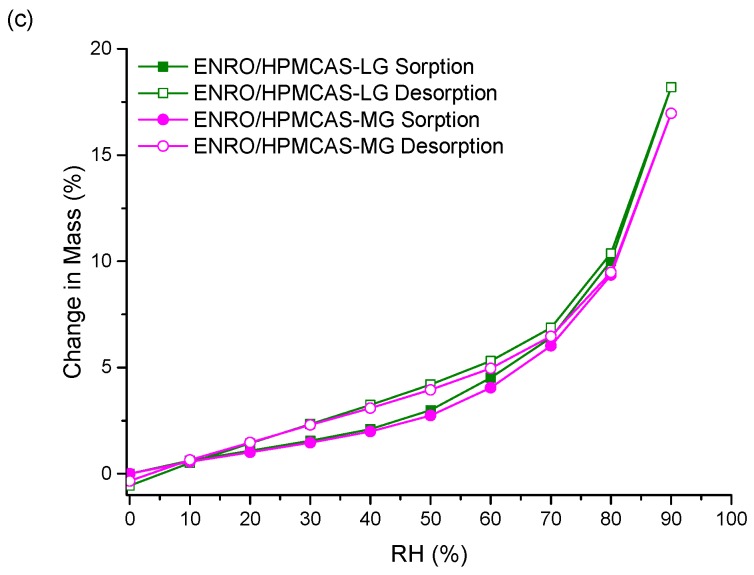
DVS isotherms of (**a**) crystalline and ball milled ENRO, (**b**) ENRO ASDs containing 40% (*w*/*w*) Eudragit L100, Eudragit L100-55, and Carbopol, and (**c**) ENRO ASDs containing 60% (*w*/*w*) HPMCAS-LG and HPMCAS-MG.

The stability of the ASDs when exposed to various humidity levels was examined by DVS. At the end of the sorption cycle, at 90% RH, ENRO absorbed only 0.13% (*w*/*w*) water. This increased to 2.9% for the ball milled drug, due to the increase in disordered material (Figure 6a). CIP also absorbed low levels of water during DVS analysis, increasing in mass by only 0.6% (*w*/*w*) [33]. PXRD analysis of the drugs at the end of the sorption studies revealed that both ENRO remained in the same solid state, with PXRD patterns matching those of the starting materials (Figure S5) [33]. In contrast to the crystalline drug, the ENRO ASDs were far more hygroscopic, absorbing 16–19% of their mass in water. Very similar levels of water uptake were observed with the CIP ASDs [9]. The higher hygroscopicity of the amorphous formulations can be explained by the random orientation of their molecules. This leads to a larger free volume and enables the penetration of water into the samples [34]. In addition, polymers are often more hygroscopic than the amorphous form of a drug, which increases the tendency of an ASD to take up moisture [35].

As can be seen in Figure 6b, the isotherms obtained with the ASDs containing Eudragit L100, Eudragit L100-55 and Carbopol were very similar in shape, with significant hysteresis. Hysteresis is commonly encountered with amorphous or porous solids, as water can absorb into the interior of the material [36]. If water diffuses into the sample bulk more quickly than it can return to the surface, then, at the same RH level, a greater amount of moisture will be present during desorption than sorption, resulting in the appearance of hysteresis. 

**Figure 7:**
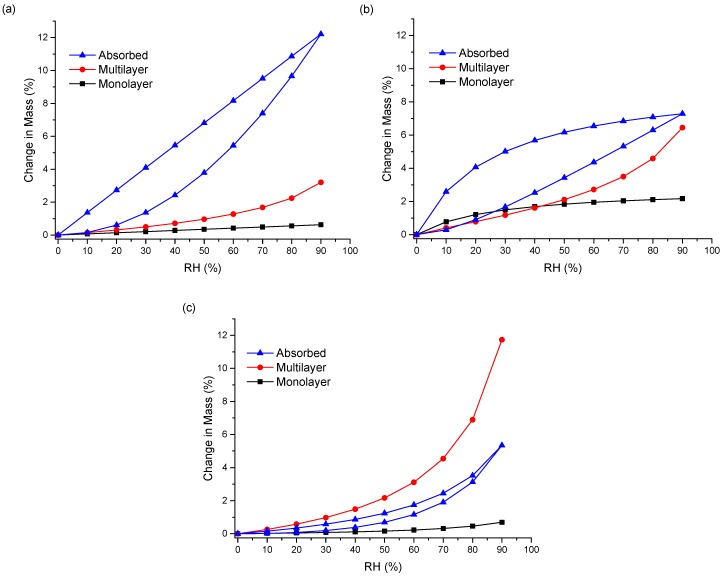
Water distribution patterns according to the Young–Nelson model in ENRO ASDs containing (**a**) Eudragit L100 40% (*w*/*w*), (**b**) Carbopol 40% (*w*/*w*), and (**c**) HPMCAS-LG 60% (*w*/*w*).

Unlike the other ASDs, the isotherms of both ENRO/HPMCAS ASDs were convex in shape with a small amount of hysteresis, suggesting that water was mainly adsorbed to the outer surfaces of these samples (Figure 6c). Therefore, the water uptake behavior of the ENRO ASDs differs depending on the polymer used. This was further examined by fitting the sorption and desorption data to the Young–Nelson equations. According to the Young–Nelson model, water can be taken up by a sample in three different ways: adsorbed as a monomolecular layer, adsorbed as a multilayer, or absorbed into the interior of the solid [22]. The parameters calculated using the Young–Nelson equations are listed in Table S1, and the isotherms obtained using this approach are shown in Figure 7 and Figure S6. The corresponding CIP ASDs were also examined for comparison (Figure S7).

As predicted from the DVS isotherms, the major water uptake mechanism of the ENRO ASDs containing Eudragit L100, Carbopol, and Eudragit L100-55 was water absorption (Figure 7a,b and Figure S6a). The small degree of absorption that occurred with the ENRO/HPMCAS ASDs confirms that they are somewhat porous, but less so than the other ASDs, as suggested by the minor hysteresis in their DVS isotherms. Unlike the other samples, the majority of water taken up by ENRO/HPMCAS ASDs was bound to their exterior surfaces as a multilayer. Multilayer formation begins at low RH levels and appears to occur simultaneously with monolayer adsorption (Figure 7c and Figure S6b). By contrast, the major water uptake mechanism for the CIP ASDs containing HPMCAS was absorption (Figure S7). This suggests that the CIP/HPMCAS ASDs are more porous than the corresponding ENRO ASDs, or the polymers may be capable of swelling to a greater degree in the former formulations. As with the ENRO ASDs, water is primarily absorbed into the interior of the CIP ASDs containing Eudragit L100, Eudragit L100-55, and Carbopol. However, the water distribution patterns obtained with the ENRO and CIP ASDs containing Carbopol differed somewhat from the others. The monolayer adsorption of these samples increased more gradually over the course of the study and was also more extensive. This may be due to the presence of more hydrophilic groups on the surface of these ASDs, which can interact with water molecules [23].

With both sets of ASDs, the highest value of E was obtained with those containing HPMCAS-LG, followed by HPMCAS-MG (Table S1). However, this constant was more than 10 times larger for the ENRO/HPMCAS samples than those containing CIP. This indicates that water molecules form much stronger and extensive interactions with the surface of these samples [37] and explains why water appears to be mainly adsorbed to the surface of these ASDs in a multilayer. The value of the regression coefficient, r, was ≥0.98 for all of the ASDs, showing that there was a good fit between the experimental and estimated values of the different parameters (Table S1). Therefore, application of the Young–Nelson model is a suitable approach for comparing the water uptake of these samples.

The permeation of water molecules into the interior of an amorphous solid can increase its free volume, resulting in a decrease in *T*_g_ [38]. Water sorption is also known to increase the molecular mobility and thus crystallization rate of amorphous substances, and to decrease the crystallization onset temperature [39]. However, despite the plasticizing effects of sorbed water, all five of the ENRO ASDs remained X-ray amorphous following DVS analysis (Figure S5). This was also the case for the corresponding CIP polymeric ASDs [9]. The high stability of these ASDs may be due to stabilizing drug–polymer interactions, the presence of which was suggested by the results of FTIR and DSC analysis. Polymers are also known to have anti-plasticizing effects and to reduce the molecular mobility of amorphous formulations, while steric hindrance from polymer chains can prevent the nucleation and crystal growth of drug molecules [8,40]. In contrast to the polymeric ASDs, amorphous CIP salts containing succinic acid or amino acids as counterions were unstable in humid environments and crystallized during DVS studies [33].

### 3.5. Solubility and Dissolution Studies

**Figure 8:**
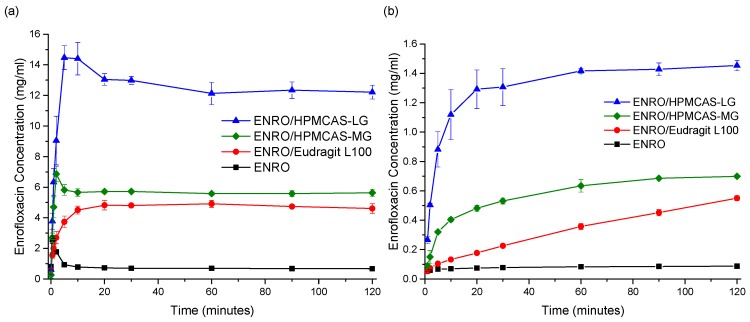
(**a**) Solubility and (**b**) dissolution studies in FaSSIF at 37 °C. The average of three experiments is plotted, ± the standard deviation.

Due to issues with clumping and viscosity, solubility studies could not be carried out accurately on the ASDs containing Eudragit L100-55 and Carbopol, and these samples were therefore excluded from further studies. The superior solubility of the remaining ASDs in FaSSIF in comparison to crystalline ENRO is clear from Figure 8a. With the pure drug, a peak in concentration was seen at 30–60 s, which then quickly fell to a constant level of approximately 0.7 mg/mL. A steep initial increase in drug concentration was also seen with the ASDs containing HPMCAS-LG and HPMCAS-MG, which peaked after 5 and 2 min, respectively. While this supersaturation then fell after 10–15 min, the concentration was still significantly higher than that obtained with crystalline ENRO. This solubility enhancement was sustained for the remainder of the study, with final concentrations of 12.2 mg/mL and 5.6 mg/mL being obtained with ENRO/HPMCAS-LG and ENRO/HPMCAS-MG, respectively. In contrast to the other samples, a more gradual increase in drug concentration was seen with ENRO/Eudragit L100, followed by a plateau after 20 min. This sample was also less soluble than those containing HPMCAS, reaching a concentration of 4.6 mg/mL after 2 h.

The dissolution behavior observed with these ASDs is similar to that described by the “spring” and “parachute” model [41]. In this model, ASDs are described as “springs”, as their high energy and lack of a crystal lattice results in rapid drug dissolution and supersaturation. However, this supersaturated state is thermodynamically unstable, and crystallization of a lower energy, less soluble form of the drug soon follows. Fortunately, excipients such as polymers may be used to inhibit or delay the precipitation of dissolved drug and thus act as “parachutes” [41]. Polymers can prevent nucleation and crystal growth via interactions with the drug, steric hindrance, and increased viscosity [42]. Although the concentration obtained with the ENRO/HPMCAS ASDs did decrease somewhat over the course of the study, the polymers present in these ASDs most likely prevented extensive crystallization of the drug in solution, enabling supersaturation to be maintained for at least 2 h. By avoiding the rapid generation of supersaturation, less nucleation and crystallization would be expected to occur with the ENRO/Eudragit L100 ASD. This was confirmed by PXRD analysis of the excess solid recovered at the end of the solubility studies. In each case, enrofloxacin hexahydrate [2] was detected; however, with ENRO/Eudragit L100, the sample was far less crystalline (Figure S8).

Similarly enhanced concentrations were obtained with the ENRO ASDs in dissolution studies in comparison to crystalline ENRO. Following 2 h, the highest concentration was achieved with ENRO/HPMCAS-LG, at 1.45 ± 0.03 mg/mL (44.8 ± 1.2% of ENRO released), followed by ENRO/HPMCAS-MG (0.70 ± 0.01 mg/mL, 40.5 ± 0.6% of ENRO released) and ENRO/Eudragit L100 (0.55 ± 0.02 mg/mL, 57.4 ± 1.8% of ENRO released). Crystalline ENRO, on the other hand, only attained 0.09 ± 0.00 mg/mL (104.7 ± 4.5% of ENRO released) over the course of the study (Figure 8b). Apart from concentration, the ASDs also differed in the shape of their dissolution profiles. With ENRO/HPMCAS-LG, the drug concentration increased quite rapidly at the start of the study and then remained fairly constant for the remainder. While a similar profile was obtained with ENRO/HPMCAS-MG, the initial drug release was more gradual than with the LG grade of polymer. As was the case in the solubility study, the final concentration obtained with ENRO/HPMCAS-MG was approximately half that of ENRO/HPMCAS-LG. However, ASDs containing different grades of HPMCAS are known to demonstrate different rates and extents of drug release, due to differences in their succinoyl and acetyl content [43]. This may affect the pH of the diffusion layer surrounding the ASD particles, or the strength of drug–polymer interactions. 

A steady, linear increase in drug concentration was observed with ENRO/Eudragit L100. As no leveling off occurred during the study, it is possible that the drug concentration would continue to rise during longer-term studies, similar to an extended release formulation. The gradual dissolution of ENRO from this ASD may be due to strong drug–polymer interactions, which could delay the dissociation and dissolution of the drug [44]. Such interactions would also explain the higher than predicted *T*_g_ of this formulation and the absence of crystallization during DSC analysis, unlike the ASDs containing HPMCAS. Alternatively, this polymer may be less soluble than HPMCAS, which would reduce the diffusion of water into the ASD and, thus, its dissolution rate.

Visible differences in the behavior of the ENRO ASD powders were also evident during dissolution studies. Both ENRO/Eudragit L100 and ENRO/HPMCAS-MG formed clumps when added to the dissolution vessels. While these eventually dissolved in the case of ENRO/HPMCAS-MG, with ENRO/Eudragit L100, they remained largely intact for the duration of the study. This would have hindered the release of the drug and reduced the surface area exposed to the dissolution medium. By contrast, no clumping was observed with ENRO/HPMCAS-LG, which enabled faster dissolution and higher concentrations of ENRO to be achieved.

From the results of this study and that of a previous investigation involving CIP, it can be concluded that ENRO is the more soluble of the two fluoroquinolones in FaSSIF. CIP was found to have a solubility of only 0.14 mg/mL in this medium [9], which is five times lower than that of ENRO. Higher drug concentrations were also obtained with the ENRO ASDs than the equivalent CIP ASDs. Similarly, ENRO has been reported to be more soluble than CIP in pH 7.4 phosphate buffer [5]. As previously mentioned, the greater solubility of ENRO may be explained by its weaker crystal lattice, which would facilitate the release of drug molecules into solution.

### 3.6. Bacterial Studies

The minimum inhibitory concentrations (MICs) and minimum bactericidal concentrations (MBCs) of ENRO and the ASDs are listed in Table 2. Values that differ significantly from those of ENRO are shown in bold. In each case, the MBC should be greater than the MIC, as a larger quantity of drug is required to bring about bacterial death rather than growth inhibition. If the ratio of MBC to MIC is ≤4, this indicates that a drug is bactericidal [45], which was the case for ENRO and the ASDs in all species of bacteria in this study. A MIC of ≤0.5 µg/mL may be considered as susceptible to ENRO, while ≥2 µg/mL indicates bacterial resistance, and 1 µg/mL is intermediate [46]. Therefore, from the results of this study, it can be concluded that *E. coli*, *S. aureus*, and *K. pneumoniae* are susceptible to ENRO, while *P. aeruginosa* is not. As was the case with CIP, *E. coli* was found to be the most susceptible of these bacteria to ENRO, having a MIC of 0.004–0.0016 µg/mL. Quite low MIC levels were also obtained in *K. pneumoniae* (0.032–0.125 µg/mL), followed by *S. aureus* (MIC 0.125–0.25 µg/mL). By contrast, much higher MIC and MBC values of 4–8 µg/mL were obtained with *P. aeruginosa*. However, the outer membrane of this bacteria is known to be far less permeable than that of *E. coli*, while fluoroquinolones are also believed to be substrates for an active efflux system within *P. aeruginosa* [47]. In each case, the MIC values obtained for ENRO in these four species agree well with those reported previously [46].

As can be seen from Table 2, the formulation of ENRO as an ASD did not significantly affect its antibacterial activity in any species of bacteria, while the MIC and MBC obtained with ENRO/HPMCAS-MG was significantly lower in *E. coli* and *K. pneumoniae* than in the pure drug. Similar results were previously obtained with CIP ASDs, whereby the MIC and MBC of CIP/HPMCAS-MG was significantly lower than crystalline CIP in all four of these species, while the MIC of CIP/HPMCAS-LG was also significantly reduced in *E. coli,* and its MBC was lower in both *E. coli* and *S. aureus*. These ASDs were also found to increase the passive transmembrane permeability of CIP [9]. Therefore, it is possible that the formulation of ENRO as an ASD with HPMCAS-MG also improved its permeability, enabling more of the drug to be transported through the bacterial cell membranes via passive diffusion.

## 4. Conclusions

In this study, ball milling was successfully used to produce several ASDs of ENRO. Despite its extra ethyl group, ENRO behaved similarly to CIP in terms of polymer compatibility, with each drug only forming X-ray amorphous ASDs with acidic polymers. The results of FTIR analysis indicate that the terminal tertiary amine of ENRO is protonated in these ASDs and forms an ionic bond with the carboxylate groups of the polymers. The high *T*_g_s of the ASDs and their resistance to crystallization during DSC analysis reinforces the suggestion that strong interactions exist between the components and formation of amorphous polymeric salts. Although the ASDs were hygroscopic, they remained X-ray amorphous during water sorption studies due to the stabilizing effects of the polymers. The ASDs also generated significantly higher drug concentrations than crystalline ENRO during solubility and dissolution testing, and these levels were sustained for the duration of the studies. As the prolongation of supersaturation is believed to increase drug absorption, the in vivo absorption of these formulations is likely to be superior to that of the pure drug. In addition, the antimicrobial activity of ENRO was not decreased by ASD formation, while it was improved by ENRO/HPMCAS-MG in *E. coli* and *S. aureus*. This study has therefore demonstrated that the formulation of ENRO as a polymeric ASD, or more accurately, an APS, can improve a number of the drug’s biopharmaceutical properties, making this an attractive formulation option.

## Figures and Tables

**Table 1:** Glass transition temperatures (*T*_g_) of ENRO and ENRO ASDs.

Sample	Experimental *T*_g_ (°C)	G-T *T*_g_ (°C)
ENRO	58.9	N/A
ENRO/Eudragit L100	109.9 ± 1.6	82.5
ENRO/ Eudragit L100-55	103.2 ± 0.2	74.0
ENRO/Carbopol	155.6 ± 0.2	71.4
ENRO/HPMCAS-LG	86.8 ± 0.4	85.6
ENRO/HPMCAS-MG	83.3 ± 0.4	85.9

**Table 2:** Minimum inhibitory concentration and minimum bactericidal concentration of enrofloxacin and ASDs in various bacteria *^a^*.

Sample	*S. aureus*	*P. aeruginosa*	*E. coli*	*K. pneumoniae*
Minimum Inhibitory Concentration (µg/mL)				
ENRO	0.25	4	0.016	0.125
ENRO/Eudragit L100	0.125–0.25	4	0.008–0.016	0.063–0.125
ENRO/HPMCAS-LG	0.125–0.25	4–8	0.008–0.016	0.063–0.125
ENRO/HPMCAS-MG	0.125	4	**0.004–0.008**	**0.032–0.063**
Minimum Bactericidal Concentration (µg/mL)				
ENRO	0.25	4	0.032	0.25
ENRO/Eudragit L100	0.25	8	0.016	0.125
ENRO/HPMCAS-LG	0.125	8	0.016	0.125
ENRO/HPMCAS-MG	0.125	4	**0.008**	**0.063**

*^a^* The values shown in bold differ significantly from those of pure crystalline ENRO.

## Supplementary Materials

The following are available online at https://www.mdpi.com/1999-4923/11/6/268/s1, Figure S1: PXRD analysis of solid dispersions formed by milling ENRO with 40–60% (*w*/*w*) HPMCAS-LG and HPMCAS-MG for 4 h at room temperature, Figure S2: FTIR peak deconvolution of (a) crystalline ENRO, (b) ball milled ENRO, (c) quench cooled ENRO, (d) ENRO/Eudragit L100, (e) ENRO/Eudragit L100-55, (f) ENRO/Carbopol, (g) ENRO/HPMCAS-LG, and (h) ENRO/HPMCAS-MG. Dotted black line: recorded spectrum; solid blue lines: deconvoluted individual Gauss peaks; and solid red line: sum of the component peaks, Figure S3: FTIR spectra of ENRO semi-crystalline solid dispersions (SD) and physical mixtures (PM) containing (a) 50% (*w*/*w*) PVP, and (b) 40% (*w*/*w*) PVA, Figure S4: HSDSC analysis of crystalline ENRO using various heating rates, Figure S5: PXRD analysis of ENRO and ENRO ASDs following DVS studies, Figure S6: Water distribution patterns according to the Young–Nelson model in ENRO ASDs containing (a) Eudragit L100-55 40% (*w*/*w*) and (b) HPMCAS-MG 60% (*w*/*w*), Figure S7: Water distribution patterns according to the Young–Nelson model in CIP ASDs containing (a) Eudragit L100 40% (*w*/*w*), (b) Eudragit L100-55 40% (*w*/*w*), (c) Carbopol 40% (*w*/*w*), (d) HPMCAS-LG 60% (*w*/*w*), and (e) HPMCAS-MG 60% (*w*/*w*), Figure S8: PXRD analysis of ENRO and ENRO ASDs following solubility studies in FaSSIF, Table S1: Parameters estimated from the Young–Nelson Model for the CIP and ENRO ASDs.
