# Supplementary material for: Fluoroquinolone Amorphous Polymeric Salts and Dispersions for Veterinary Uses
Source: Pharmaceutics. 2019 Jun 9;11(6):268. doi: 10.3390/pharmaceutics11060268 (PMC6631417; doi:10.3390/pharmaceutics11060268)
Supplement: Supplementary file 1 [file pharmaceutics-11-00268-s001.pdf]

Supporting Information

Fluoroquinolone amorphous polymeric salts and dispersions for veterinary uses

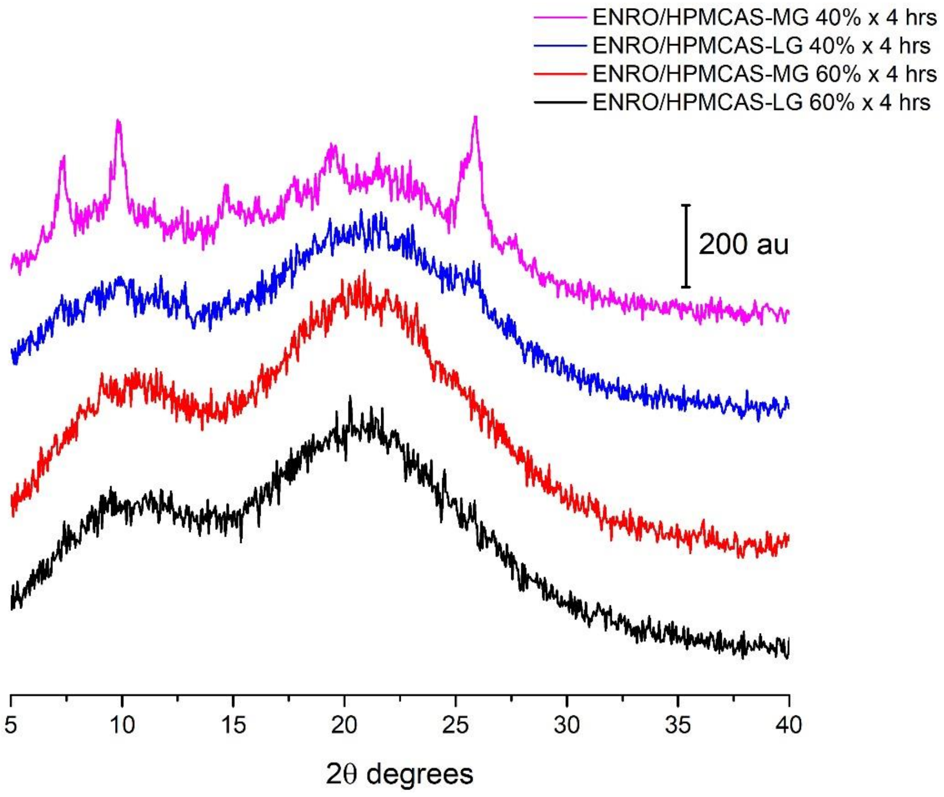

Figure S1. PXRD analysis of solid dispersions formed by milling ENRO with 40–60% (w/w) HPMCAS-LG and HPMCAS-MG for 4 hours at room temperature.

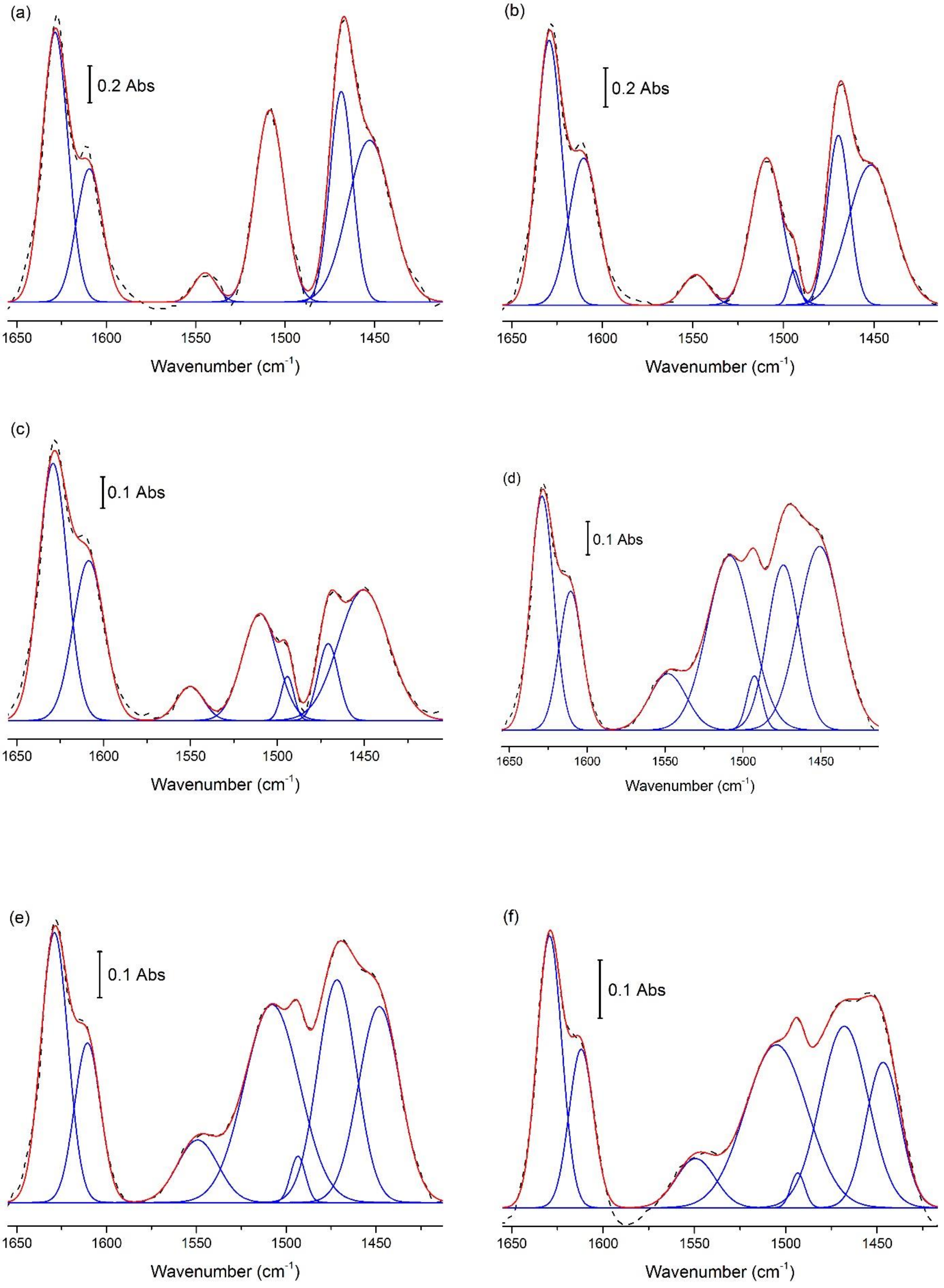
(a)
0.2 Abs
1650
1600
1550
1500
1450
Wavenumber (cm-1)
(b)
0.2 Abs
1650
1600
1550
1500
1450
Wavenumber (cm-1)
(c)
0.1 Abs
1650
1600
1550
1500
1450
Wavenumber (cm-1)
(d)
0.1 Abs
1650
1600
1550
1500
1450
Wavenumber (cm-1)
(e)
0.1 Abs
1650
1600
1550
1500
1450
Wavenumber (cm-1)
(f)
0.1 Abs
1650
1600
1550
1500
1450
Wavenumber (cm-1)

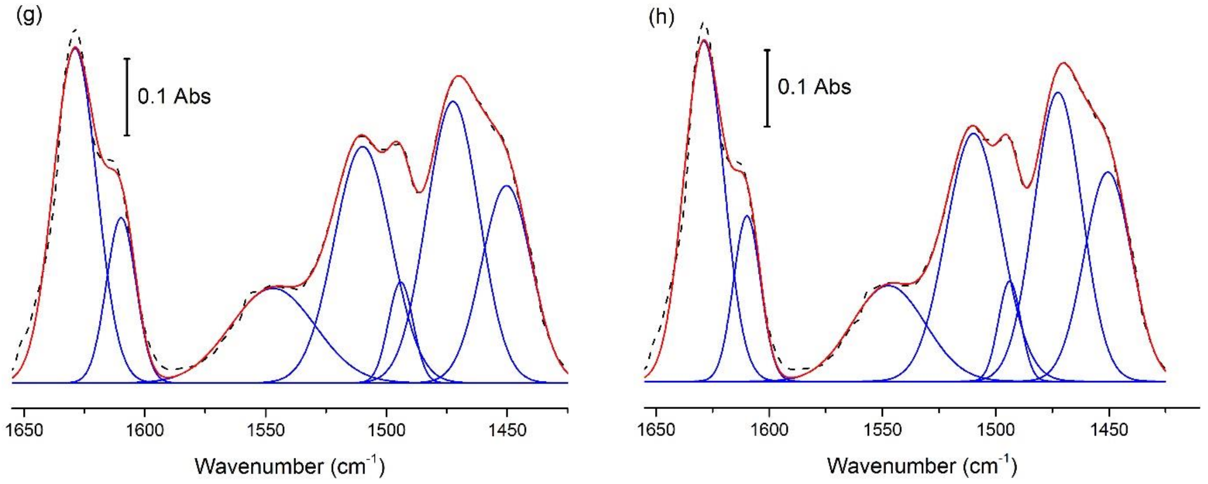

Figure S2. FTIR peak deconvolution of (a) crystalline ENRO (b) ball milled ENRO (c) quench cooled ENRO (d) ENRO/Eudragit L100 (e) ENRO/Eudragit L100-55 (f) ENRO/Carbopol (g) ENRO/HPMCAS-LG and (h) ENRO/HPMCAS-MG. Dotted black line: recorded spectrum; solid blue lines: deconvoluted individual Gauss peaks; and solid red line: sum of the component peaks.

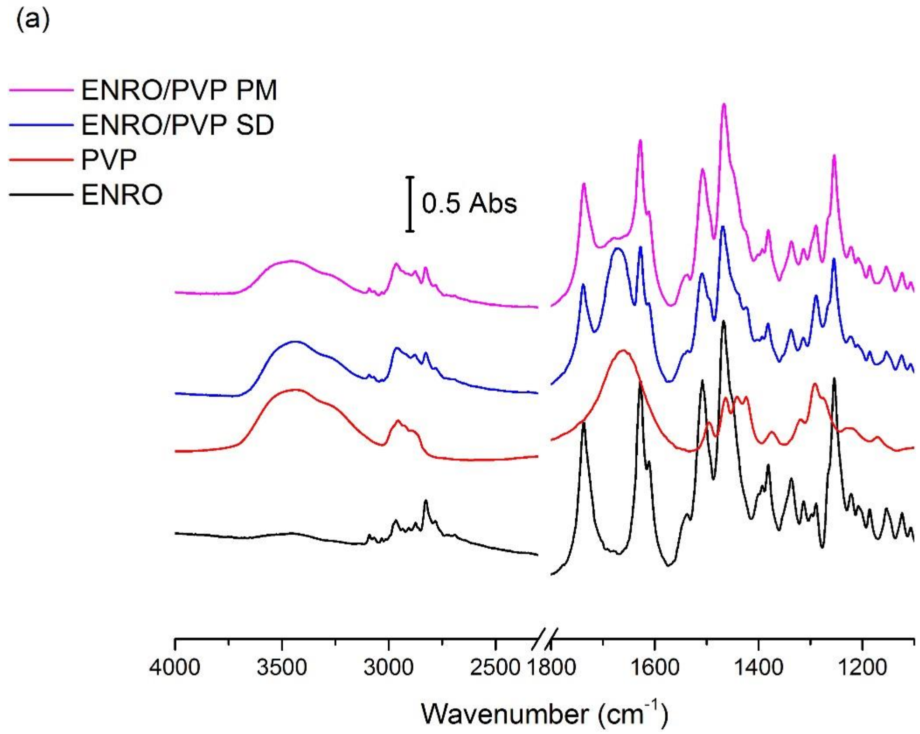

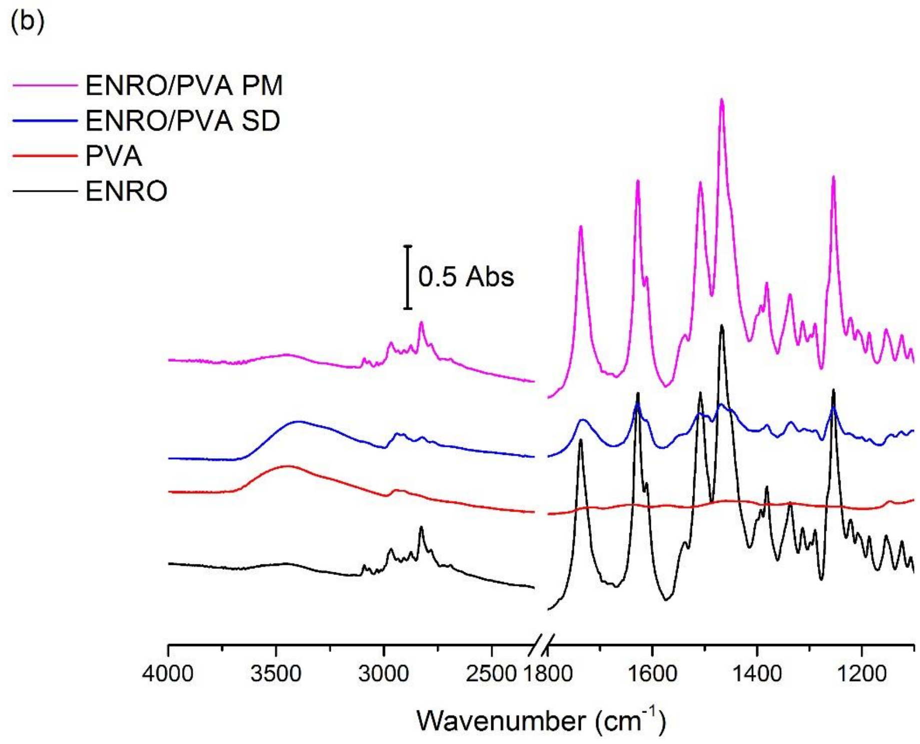

Figure S3. FTIR spectra of ENRO semi-crystalline solid dispersions (SD) and physical mixtures (PM) containing (a) 50% (w/w) PVP, and (b) 40% (w/w) PVA.

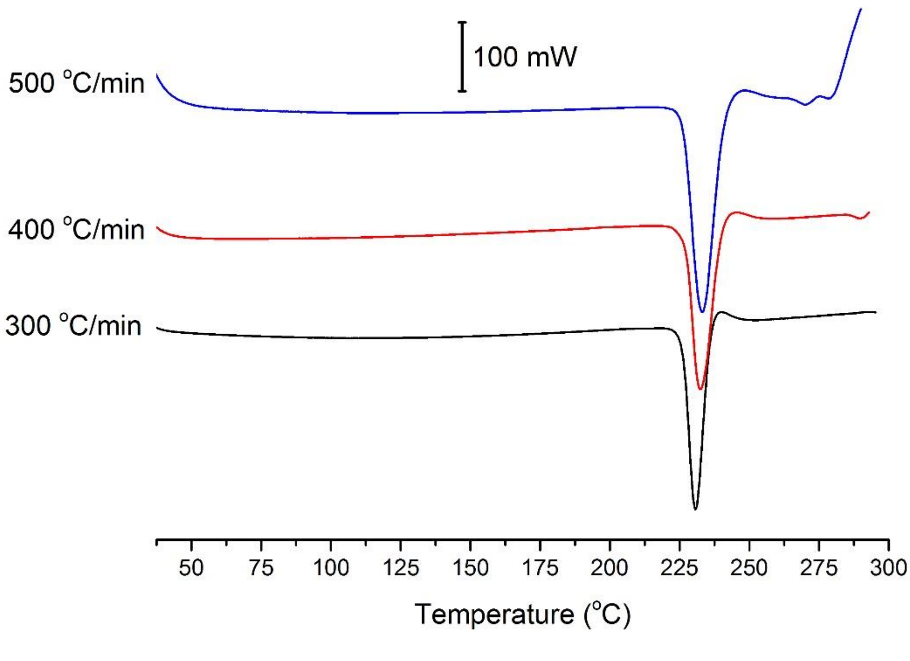

Figure S4. HSDSC analysis of crystalline ENRO using various heating rates.

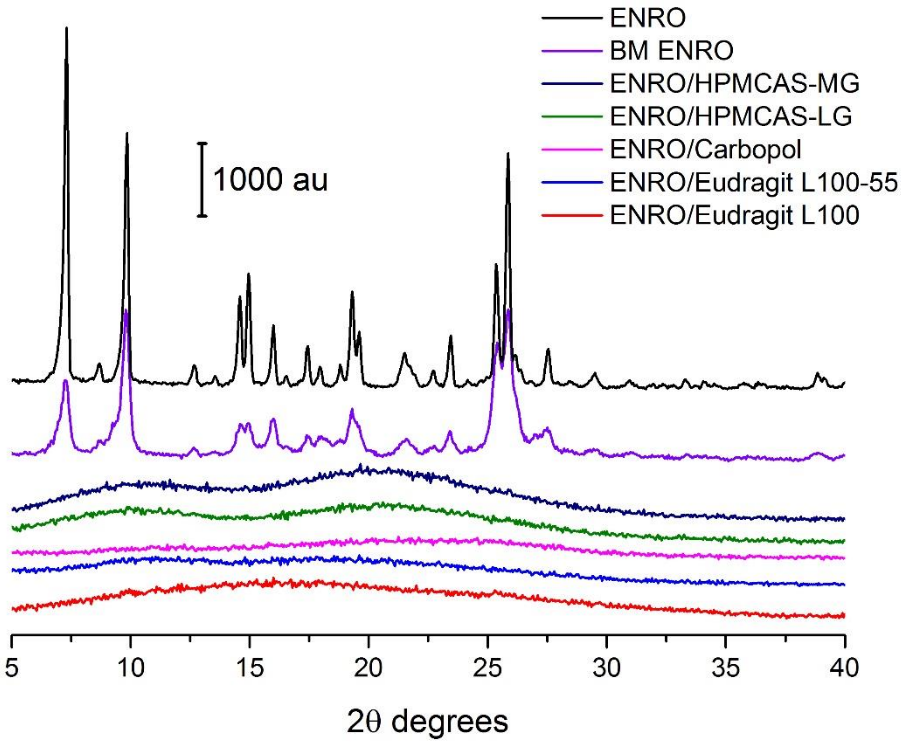

Figure S5. PXRD analysis of ENRO and ENRO ASDs following DVS studies.

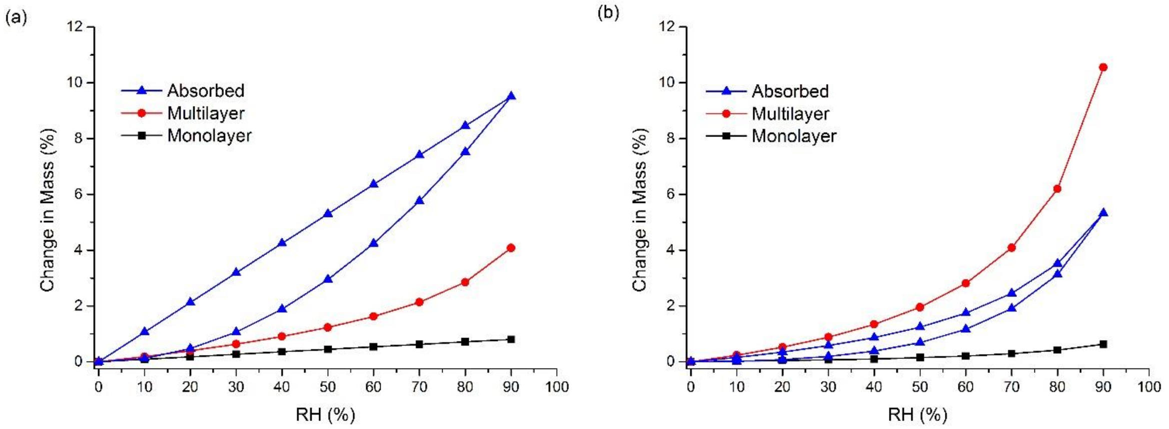

Figure S6. Water distribution patterns according to the Young-Nelson model in ENRO ASDs containing (a) Eudragit L100-55 40% (w/w) and (b) HPMCAS-MG 60% (w/w).

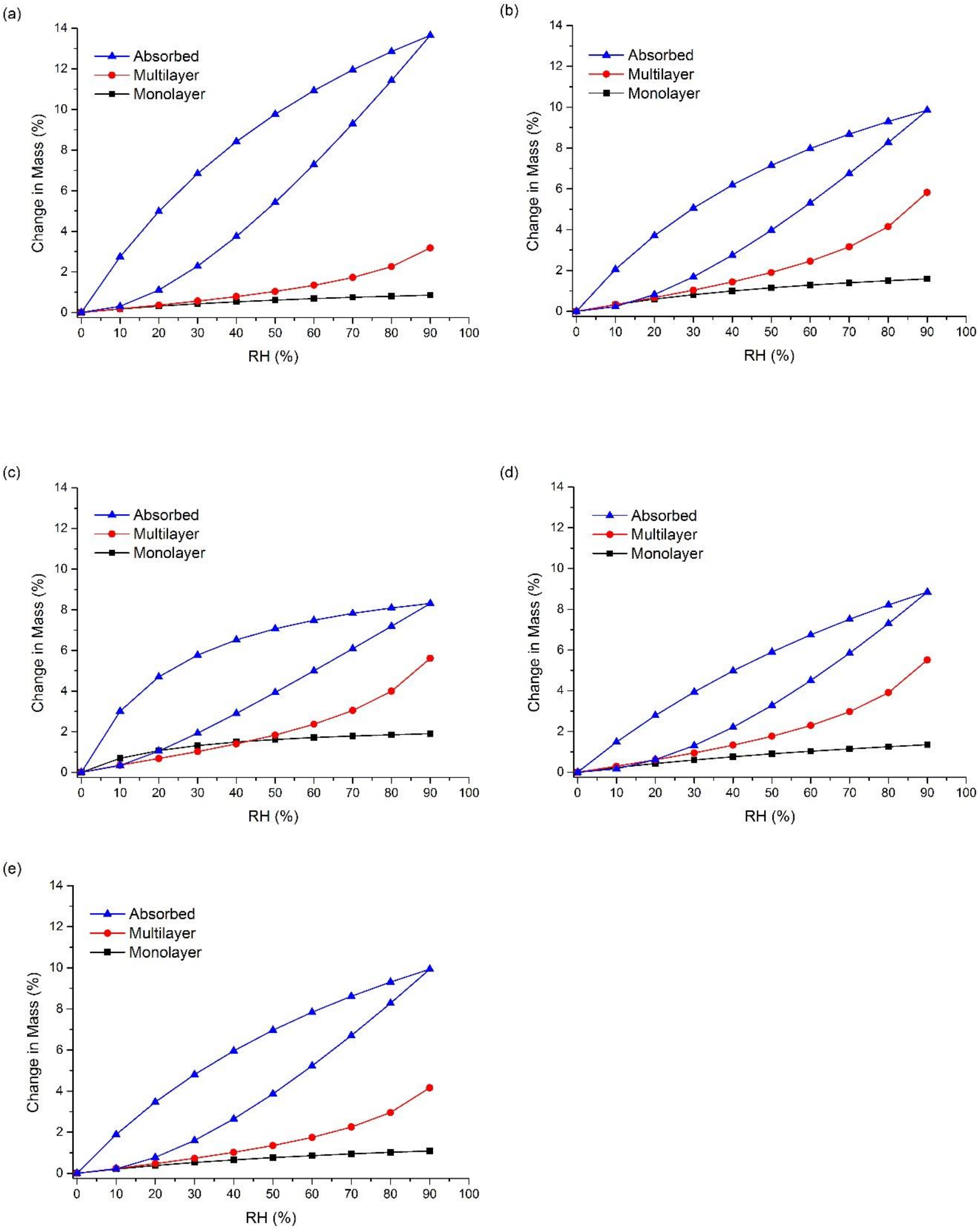

Figure S7. Water distribution patterns according to the Young-Nelson model in CIP ASDs containing (a) Eudragit L100 40% (w/w) (b) Eudragit L100-55 40% (w/w) (c) Carbopol 40% (w/w) (d) HPMCAS-LG 60% (w/w) and (e) HPMCAS-MG 60% (w/w).

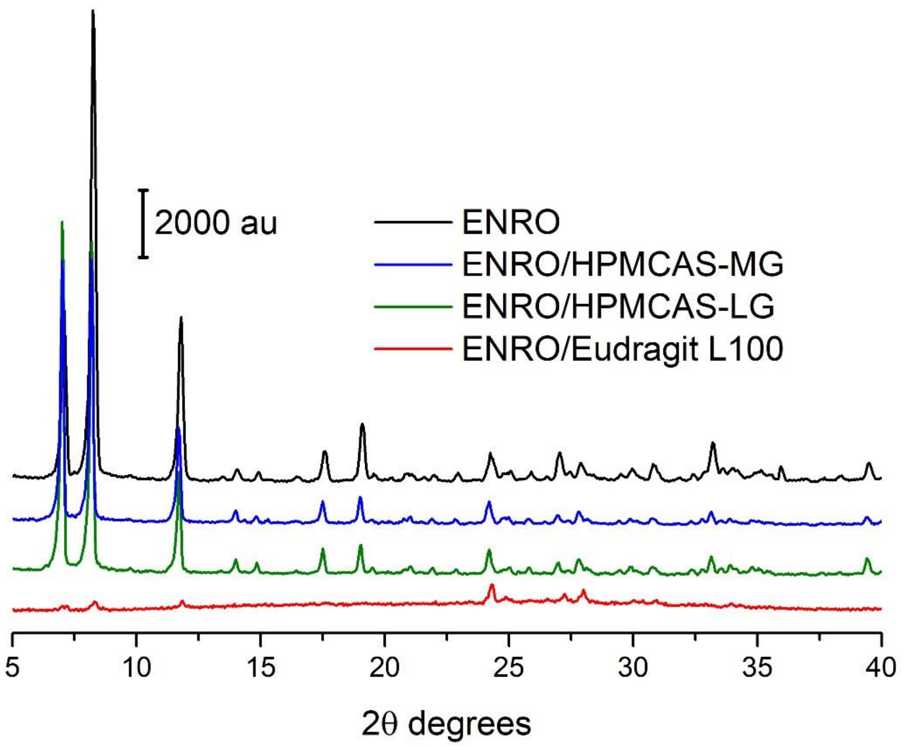

Figure S8. PXRD analysis of ENRO and ENRO ASDs following solubility studies in FaSSIF.

Table S1. Parameters Estimated from Young-Nelson Model for the CIP and ENRO ASDs

**ASD**	**A**[a]	**B**[b]	**E**[c]	**r**[d]
ENRO/Eudragit L100	0.007	0.151	0.990	0.981
ENRO/Eudragit L100-55	0.009	0.117	0.990	0.978
ENRO/Carbopol	0.022	0.083	0.209	0.998
ENRO/HPMCAS-LG	0.012	0.101	6.330	0.991
ENRO/HPMCAS-MG	0.011	0.100	6.281	0.989
CIP/Eudragit L100	0.009	0.160	0.471	0.997
CIP/Eudragit L100-55	0.017	0.115	0.447	0.991
CIP/Carbopol	0.019	0.094	0.203	0.999
CIP/HPMCAS-LG	0.014	0.105	0.597	0.995
CIP/HPMCAS-MG	0.011	0.117	0.507	0.996

[a]A: fraction of adsorbed water. [b]B: fraction of absorbed water. [c]E: equilibrium constant. [d]r: correlation coefficient.
